# Supplementary figures and images for: Comparative analysis of SNP candidates in disparate milk yielding river buffaloes using targeted sequencing
Source: PeerJ. 2016 Jul 7;4:e2147. doi: 10.7717/peerj.2147 (PMC4941740; doi:10.7717/peerj.2147)

Supplementary figure 1

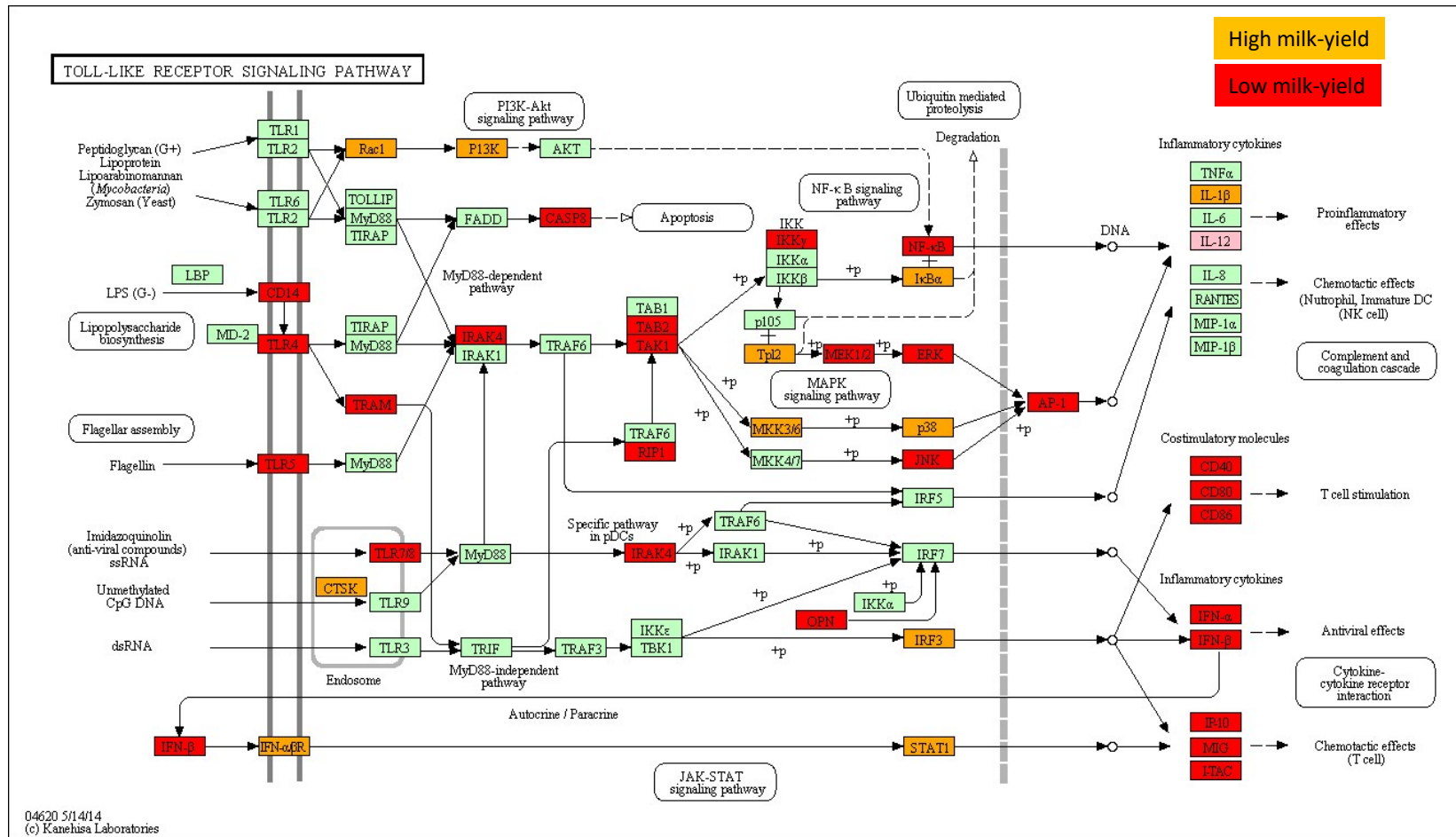

Supplementary figure 1 : Toll-like receptor signaling

Supplement: Supplemental Information 5 [file peerj-04-2147-s005.pdf]

Supplementary figure 2

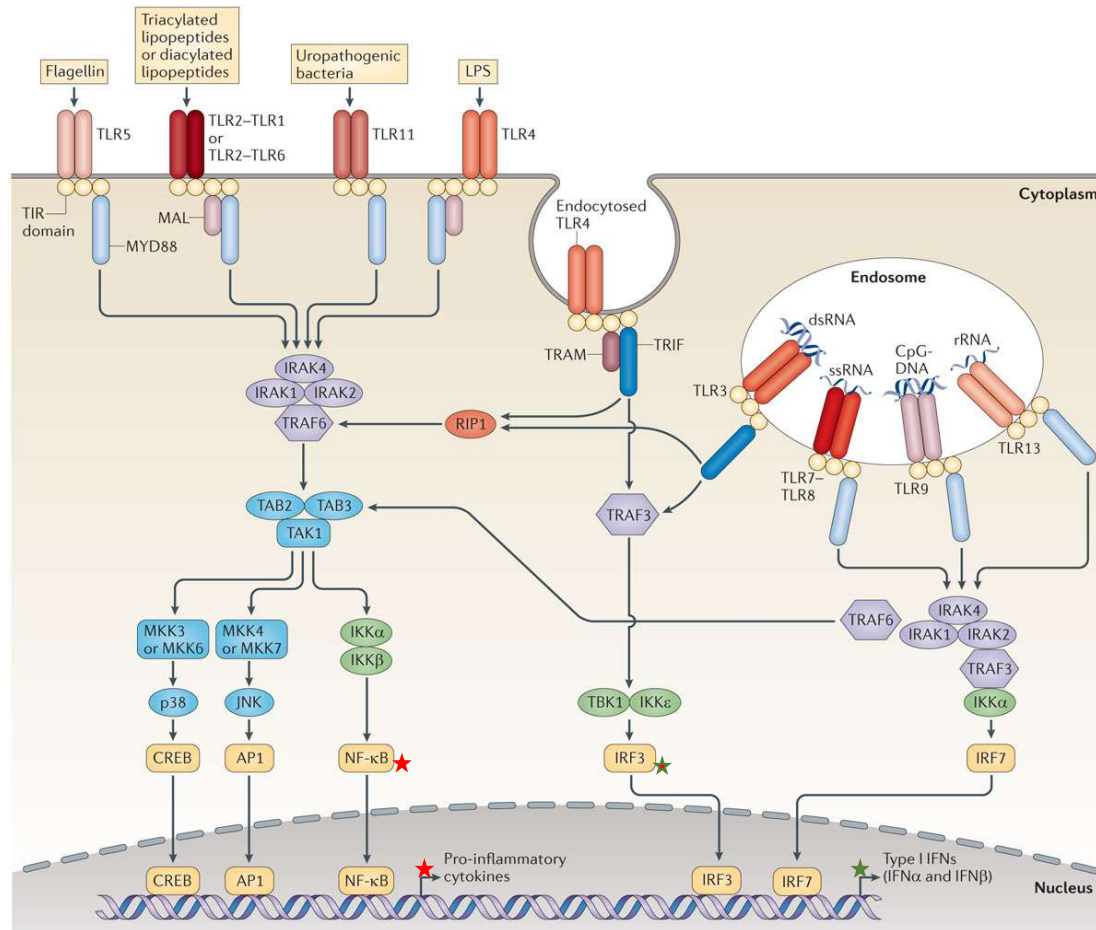

O'Neill et. al., 2013  
Nature Reviews | Immunology

Supplement: Supplemental Information 6 [file peerj-04-2147-s006.pdf]

### Supplementary figure 3 : MAPK signaling pathway

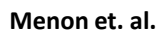

Supplement: Supplemental Information 7 [file peerj-04-2147-s007.pdf]
